# Emergent Interpolymer Interactions in Flowing Polymer Solutions

**DOI:** 10.1021/acsmacrolett.6c00010

**Published:** 2026-02-14

**Authors:** Vincenzo Calabrese

**Affiliations:** POLYMAT, Rheology and Advanced Manufacturing group, University of the Basque Country UPV/EHU, Avenida Tolosa 72, Donostia-San Sebastian 20018, Spain

## Abstract

Polymer solutions are ubiquitous across biological, healthcare, and industrial processes. When subjected to sufficiently high deformation rates, polymer chains transition from their equilibrium coiled state to a flow-induced stretched configuration, giving rise to distinctive flow behaviors often associated with stringiness, sliminess, and stickiness. While interactions between coiled polymers at equilibrium are relatively well understood, those between flow-stretched chains continue to raise fundamental questions, introducing uncertainty in how they should be accounted for. Despite decades of research, experimental efforts to infer these emergent interactions under flow have proven challenging, often yielding contrasting interpretations of their role. In this Viewpoint, we revisit classical experiments through the lens of recent studies. We outline the principal frameworks used to describe the onset of interpolymer interactions under flow and offer a unified perspective on the conditions under which such interactions are likely to emerge.

## Graphical abstract

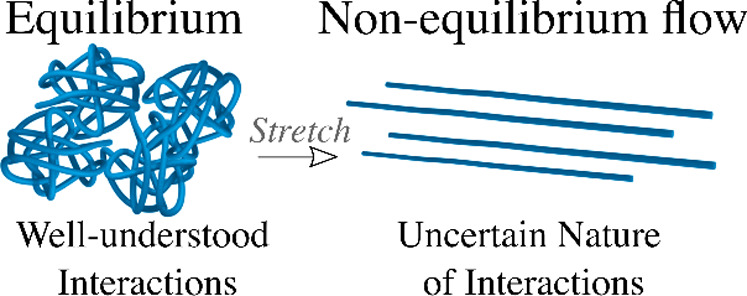

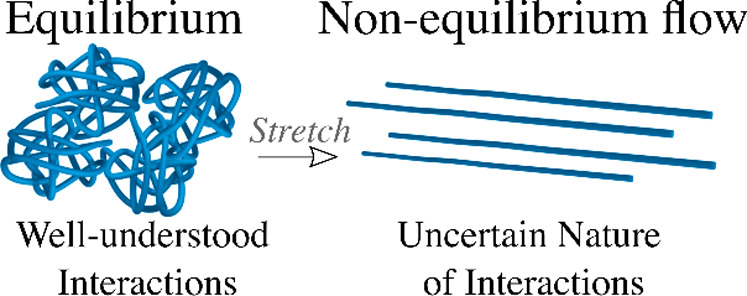

## Introduction

Solutions of long-chain polymers, such as DNA, proteins, polysaccharides, or synthetic polymers, are ubiquitous in everyday life, appearing in fluids like ink, mucus, saliva, and various food products. The functionality of these solutions depends critically on how they respond when subjected to deformation. For simple Newtonian fluids, the flow behavior can be predicted accurately over a wide range of conditions because they lack an evolving microstructure and exhibit a linear stress response to the rate of deformation. In contrast, for complex fluids such as polymer solutions, predicting the flow behavior is far more challenging, as their structural components can store elastic energy and undergo time- and history-dependent dynamics, resulting in a nonlinear stress response. This challenge has prompted work in mainly two complementary research threads. The first focuses on the experimental assessment of polymers and polymeric flow behavior, while the second aims to refine mathematical frameworks for its description. Mathematical frameworks, aimed at describing real polymer solutions, root their assumptions in experimental evidence. On the other hand, experimental work corroborates or disproves the proposed models and provides new evidence necessary to build refined versions.^1,2^ Although experimental tools to assess polymer microstructure and dynamics under flow continuously improve, there are still experimental challenges and open questions.

In this Viewpoint, we examine experimental efforts devoted to answering one of these remaining open questions. Specifically we ask how do emergent interpolymer interactions under flow affect the microstructure and flow behavior of polymer solutions? We aim to clarify under which conditions interpolymer interactions become significant for flow-stretched chains compared to their equilibrium state. Our focus is on concentrations up to the semidilute regime, where interpolymer interactions at equilibrium are expected to be relatively mild. For a complementary perspective on stretched polymers in the melt state, we refer the reader to the review by Huang.^3^ Given the breadth of the topic, we focus our discussion on steady and quasi-steady shear and extensional flows because these represent the simplest flow configurations and allow for a clearer interpretation of polymer dynamics. We also discuss polymer behavior in capillary-driven extensional flows, as this type of flow has been widely used as a *rheometric* tool to infer interpolymer interactions under extensional deformation across the dilute and semidilute regimes.^4^ With regard to the type of polymer solutions, particular emphasis is placed on studies involving monodisperse linear double-stranded DNA and low-polydispersity polystyrene solutions. The study of nearly monodisperse polymer samples facilitates straightforward cross-comparison between experiments conducted by different research groups. In particular, DNA has been widely used by the rheology community because of its ease of fluorescent labeling, enabling the unique ability to track polymer dynamics at the single-molecule level.^5–7^ In contrast, polystyrene has been extensively employed because of its commercial availability at high molecular weights, low polydispersity, and relatively high intrinsic birefringence, the latter facilitating birefringence measurements at relatively low concentrations.^7^

We begin by briefly outlining key concepts of the behavior of polymers in thermodynamic equilibrium before moving on to the discussion of polymers in homogeneous shear and extensional flows.

## Equilibrium

At thermodynamic equilibrium, flexible polymers adopt a coil-like conformation with a coil size that, for a given polymer and molecular weight (*M*_w_), is determined by the polymer’s backbone rigidity and solvent quality.^1^ Polyelectrolytes (i.e., charged polymers), such as DNA and hyaluronic acid, exhibit a more rigid backbone in salt-free solvents compared to neutral polymers such as polystyrene and poly­(ethylene oxide), due to electrostatic repulsions between monomers.^1,7,8^ The rigidity of the chain affects the compactness of the equilibrium polymer coil, which in turn determines the level of intrachain hydrodynamic interaction. For instance, high-*M*_w_ neutral polymers form so-called nondraining coils, in which each monomer of the chain is hydrodynamically shielded from external flow fields.^7^ In contrast, more rigid polyelectrolytes tend to adopt open, free-draining coil configurations, in which the monomers are more exposed to external flow fields.^7^ Importantly, polyelectrolytes and neutral polymer chains at equilibrium are governed by different dominant intramolecular interactions. In salt-free polyelectrolyte solutions, electrostatic interactions and counterion condensation play a central role, whereas in neutral polymers, solvent quality and excluded-volume interactions dominate.^8,9^ In the context of polymers under flow, conformational differences at equilibrium are typically parametrized by the polymer’s extensibility defined as *L* = *l*_c_/*X*_0_, with *l*_c_ the polymer contour length and *X*_0_ the equilibrium coil size. Polyelectrolytes in salt-free media are generally considered semiflexible polymers with $L∼O\left(10^{1}\right)$ in between rigid rod-like polymers *L* = 1 and flexible polymers with $L≳O\left(10^{2}\right)$ (Figure 1).^10,11^ However, while *L* is a convenient and widely used parameter, it does not explicitly encode the physical origin of a given polymer conformation. For instance, a low-*M*_w_ neutral polymer can yield an *L* analogous to that of a high-*M*_w_ salt-free polyelectrolyte, yet their intrachain interactions are fundamentally different.

**Figure 1:**
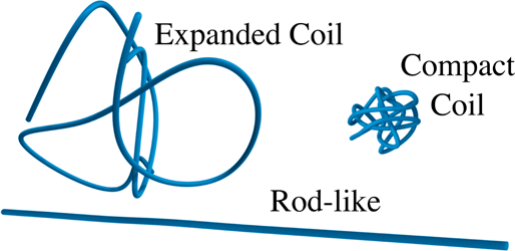
Schematics of distinct polymer conformations at equilibrium. A compact coil typical of flexible polymers with $L≳O\left(10^{2}\right)$ , a more expanded coil characteristic of semiflexible polymers with $L∼O\left(10^{1}\right)$ , and a rigid, rod-like conformation with *L* = 1.

For polymer solutions at the equilibrium, molecular weight *M*_w_, coil size *X*_0_, polymer concentration *c*, and the hydrodynamic interactions are interconnected factors determining the macroscopic viscoelasticity of these fluids.^1^ The strong coupling between microstructure, at the scale of individual polymer chains, and the resulting mechanical response of the fluid (such as viscosity and elasticity) is generally referred to as the structure–property relationship.^12,13^ This relationship has allowed experimentalists to indirectly infer equilibrium information about polymer conformations and interactions by analyzing the fluid response under relatively small deformation rates. A widely used and effective approach that exploits this structure–property relationship to assess different regimes of interpolymer interactions is based on the extrapolation of the zero-shear rate viscosity η_0_; that is, the viscosity in the limiting case of a negligibly small deformation rate so that the polymer preserves its equilibrium conformation. Taking into account the solvent viscosity η_s_ and plotting the polymeric viscosity η_p_ = η_0_ – η_s_ at different polymer concentrations *c*, several regimes are typically observable on a double logarithmic scale. An example of the η_p_ trend for monodisperse λ-DNA is shown in Figure 2(a). Generally, the concentration at which the first transition from the scaling η_p_ ∼ *c* ^n^ to a steeper concentration dependence occurs is identified as the overlap concentration c*. As the polymer concentration increases further, interpolymer interactions strengthen, and the solution eventually enters the entangled regime, characterized by a distinct η_p_ scaling.^15,19^ The overlap concentration c* has a particular meaning because it is the point at which polymer chains begin to make contact with each other and thus is informative of the onset of interpolymer interactions. The overlap concentration c* can be estimated by considering the polymer volume required to fill the available solution space as

1 $$c^{*}=\frac{M_{w}\phi_{\mathrm{pf}}}{\left(\phi_{\mathrm{sph}}N_{A}\right)}$$

where ϕ_pf_ is the packing fraction, *N*_A_ is the Avogadro’s number, and ϕ_sph_ = π*R*_g_^3^4/3 is the sphere-equivalent volume occupied by a polymer at equilibrium with radius of gyration *R*_g_.^20^ Considering ϕ_pf_ = 0.52 for simple cubic packing and an estimated *R*_g_ = 500 nm for λ-DNA at low ionic strength, we obtain c* ≈ 40 μg/mL, which is generally in agreement with the concentration at which the first transition of η_p_ scaling occurs.^15,21^

**Figure 2:**
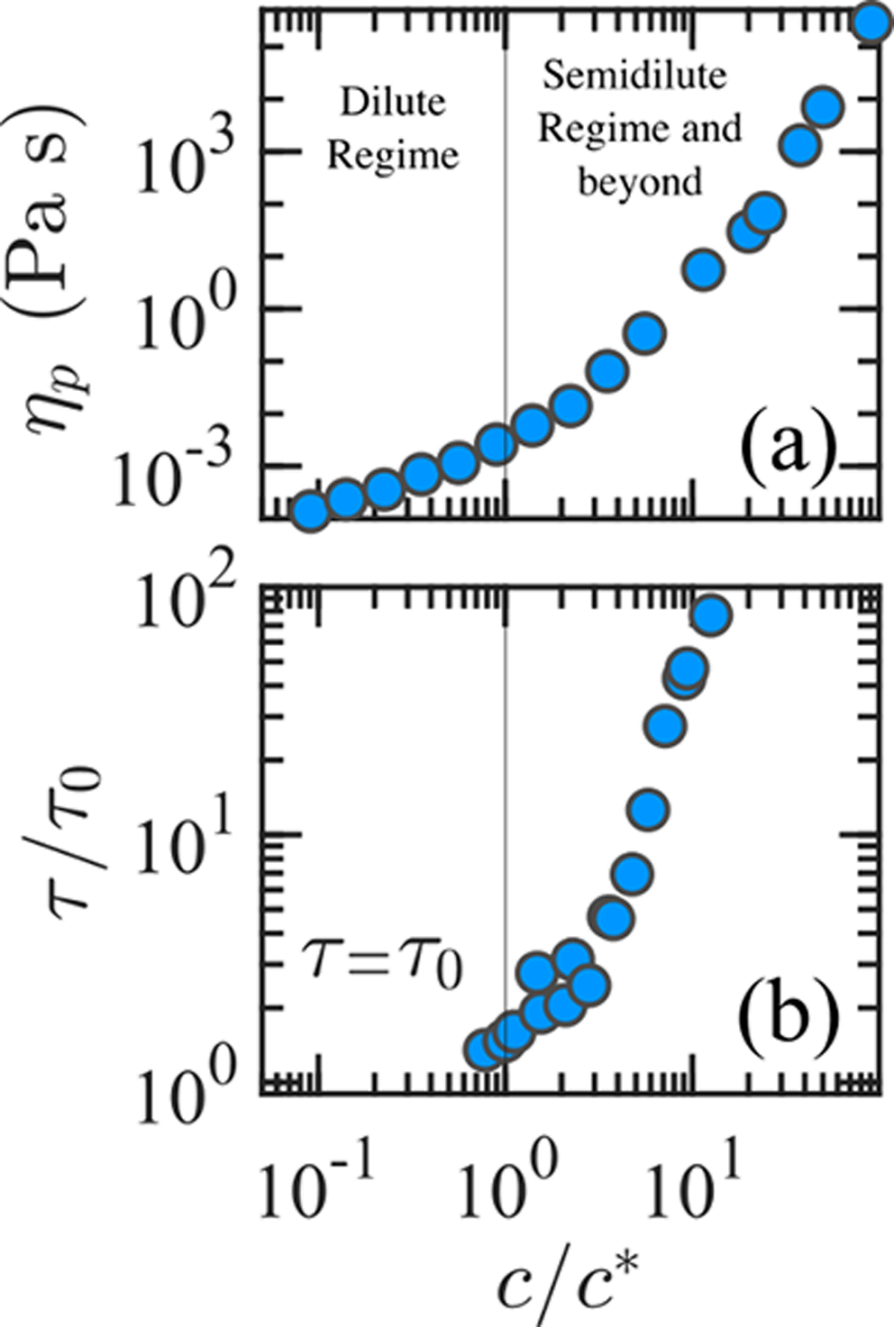
(a) Polymeric viscosity η_p_ for λ-DNA solutions. Data in (a) include λ-DNA in a buffer at relatively high ionic strength (from Pan et al.,^14^) and at moderate ionic strength (from Banik et al.,^15^). The overlap concentrations used to normalize the data are c* = 88 and c* = 40 μg/mL for the high and moderate ionic strength solutions, respectively. (b) Relaxation time τ, normalized by its infinite-dilution value τ_0_, as a function of concentration, as reported by Zhou and Schroeder,^16^ including data from Pan et al.^14^ and Hsiao et al.^17^ In panels (a) and (b), data sets from the cited sources are plotted together for clarity. Data were extracted from the cited sources using WebPlotDigitizer.^18^

Alternatively, the level of interpolymer interactions at equilibrium is commonly gauged by monitoring the longest polymer relaxation time τ as a function of concentration, as shown in Figure 2(b) for λ-DNA solutions.^16^ The longest polymer relaxation time is a material property that can be determined using various approaches, including classical rheometry and more recent microfluidic-based techniques.^22,23^ While different measurement approaches for τ are generally expected to yield equivalent results, in practice this is challenging, as each method may probe different relaxation mechanisms, and it is often unclear which modes are being measured. It is experimentally and theoretically well established that, for *c*/c* ≲ 1, τ is independent of concentration and takes the limiting value τ_0_, as the dynamics of each polymer chain are not influenced by neighboring polymers.^21^ However, as *c* increases beyond c*, τ increases and follows different scaling regimes (Figure 2(b)).

## Under Flow

Polymer coils unravel and stretch under specific flow conditions. In general, they undergo a coil-to-stretch transition when the strength of the applied flow, whether shear or extensional, is sufficient to overcome the entropic elasticity of the polymer chain.^1,24^ Investigation of polymer dynamics under flows usually requires control or knowledge of the flow type, flow strength, and the time that polymer chains are exposed to a given specific flow.

In laboratory settings, the simplest and most common flow types used to study polymer dynamics are homogeneous shearing and extensional flows. In shearing flows, the shear rate γ̇ is defined as the velocity gradient perpendicular to the flow direction γ̇=∂v/∂y where *v* is the velocity along the flow direction and *y* is the coordinate perpendicular to the flow.^24^ Shearing flows are composed of an equal amount of rotation and extensional components. In contrast, extensional flows consist exclusively of the extensional component, where fluid elements experience pure stretching or compression along the flow direction, without any rotational component. In extensional flows, the extension rate is defined as the velocity gradient along the streamwise direction ε̇=∂v/∂x , where *x* is the coordinate in the flow direction.^24^ Knowledge of the magnitude of γ̇ and ε̇ is typically compared against the longest polymer relaxation time τ via the Weissenberg number Wi≡τε̇≡τγ̇ , quantifying the strength of the deformation rate relative to the polymer propensity to undergo nonequilibrium dynamics. For *Wi* ≳ 0.5 polymers are expected to enter the out-of-equilibrium state and eventually start to unravel. It is important to note that, for a chemically identical polymer, the relaxation time τ depends on *M*_w_, backbone rigidity, solvent viscosity, and, as shown in Figure 2(b), at *c* ≳ c*, it also depends on polymer concentration. It is perhaps intuitive that when a specific *Wi* ≳ 0.5 is imposed, the polymer does not instantaneously adopt a steady conformation but instead requires a finite residence time *t*_res_. Generally, for *t*_res_ ≫ τ the polymer is considered to have reached its steady conformational state. In extensional flows, it is common to evaluate the accumulated fluid strain (or Henky strain) $\varepsilon =t_{\mathrm{res}}\mathrm{\varepsilon ̇}$ or the accumulated macromolecular strain $\varepsilon_{\mathrm{mol}}=t_{\mathrm{res}}\left(\mathrm{\varepsilon ̇}-{\mathrm{\varepsilon ̇}}_{c}\right)$ .^25^ The accumulated macromolecular strain ε_mol_ is particularly useful because it takes into account that a polymer can accumulate deformation only above a critical extension rate ${\mathrm{\varepsilon ̇}}_{c}\approx 0.5/\tau$ . The *t*_res_ at which the condition exp­(ε_mol_) = *L* is satisfied provides a lower-bound estimate of the minimum residence time required for the polymer to reach the fully stretched state.^25^

An important consequence of flow-induced stretching of polymer chains is the increase in hydrodynamic drag as the polymer extends, ultimately leading to a drag coefficient for stretched chains, ζ_*s*_, that exceeds that of the equilibrium coil, ζ_*c*_. The ratio ζ_s_/ζ_c_ increases significantly with the polymer’s extensibility *L*. As a result, highly flexible polymers exhibit relatively high values of ζ_s_/ζ_c_, which implies a drastic increase in hydrodynamic drag upon stretching compared to their equilibrium state.^7^ Based on the magnitude of the ratio ζ_s_/ζ_c_, a phenomenon known as coil-to-stretch hysteresis has been predicted^26–28^ and subsequently experimentally observed by direct observation of relatively large DNA with *l*_c_ ≈ 1.3 mm.^29^ In the presence of coil-to-stretch hysteresis, a polymer chain subjected to the same *Wi* can exhibit two stable levels of stretching, one more stretched than the other, depending on whether its initial state is coiled or highly stretched, or more generally, on its deformation history (see sketch in Figure 3(a)). Experiments and simulations suggest that, for nominally dilute solutions, a ratio ζ_s_/ζ_c_ ≳ 4.5 is required for the coil-to-stretch hysteresis to occur.^17,29^ This implies that, for a given polymer type, relatively high-*M*_w_ polymers are expected to display coil-to-stretch hysteresis, whereas low-*M*_w_ polymers are not, as illustrated in Figure 3(a) and (b), respectively. Prabhakar et al. theoretically investigated the effect of polymer concentration on the coil-to-stretch hysteresis.^30^ Interestingly, their work suggested that the coil-to-stretch hysteretic window widens with increasing concentration for dilute solutions up to approximately c*, and then progressively narrows, eventually disappearing as the polymer concentration increases beyond c*.^30^

**Figure 3:**
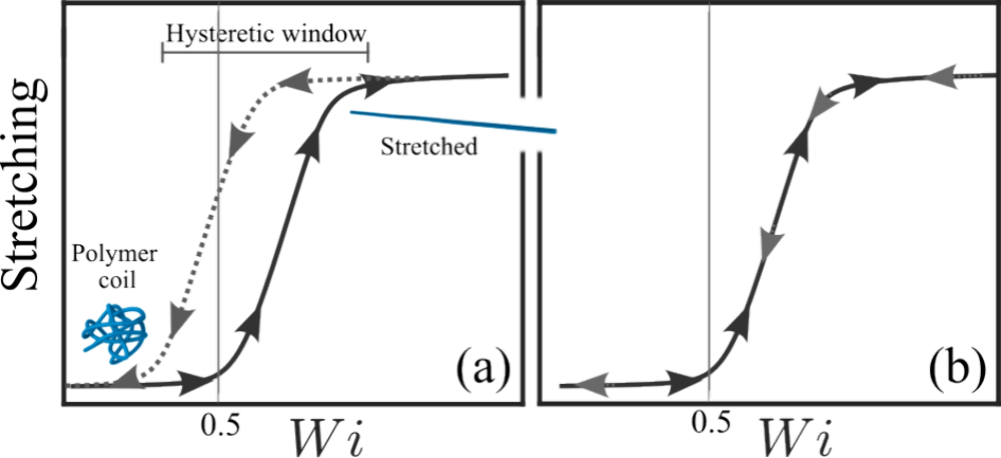
Schematic illustration of the extent of polymer stretching as a function of the Weissenberg number (*Wi*) for (a) a polymer exhibiting coil-to-stretch hysteresis and (b) one that does not. Arrows indicate the increasing or decreasing *Wi* during the cycle.

## Shear Flows

Pioneering experiments on DNA in shear flow were conducted in 1999 by Smith et al.^33^ In these experiments, the stretching and alignment dynamics of fluorescently labeled DNA molecules were probed in the velocity-vorticity plane for isolated DNA at concentrations regime significantly below c*. The results revealed that DNA does not adopt a single stable conformation in steady shear flows but instead exhibits pronounced fluctuations in polymer extension and tumbling due to the rotational component of shear flow. Later, direct observations of DNA in the velocity-gradient plane revealed the periodicity of the tumbling events (Figure 4(a)).^31^ This periodicity was evident by tracking, at a given shear rate, the fractional polymer extension α = *X*/*l*_*c*_, where *X* is the end-to-end polymer distance, over time (Figure 4(b)). Steady-state experiments (i.e., when the mean fractional extension ⟨α⟩ reaches a steady value) revealed that the stretching dynamics of λ-DNA in shear flow at comparable *Wi* were independent of polymer concentration (up to 6c*).^32^ This was strongly supported by the probability distribution function (PDF) of polymer extension (Figure 4(c)), the steady-state average fractional polymer extension ⟨α⟩ = ⟨*X*⟩/*l*_c_ (Figure 4(d), angle brackets denote time-averaged quantities), and the temporal fluctuations in molecular extension (not shown here), all of which remained independent of concentration. While the aforementioned analyses focused on steady-state polymer conformations, DNA dynamics in transient start-up experiments, where molecules are suddenly exposed to an increased shear rate, also exhibited concentration-independent behavior.^32,35^ These experiments may suggest that once the λ-DNA enters a nonequilibrium extended state, interpolymer interactions mediated by direct polymer–polymer contacts become less significant. As a result, within the concentration range investigated, the polymers behave essentially as if they were dilute. Brownian simulations accounting for hydrodynamic interactions in the concentration range 0 < *c*/c* ≤ 2 for the steady-state fractional extension of λ-DNA as a function of *Wi* showed good agreement with experimental observations (see black circles in Figure 4(d)).^34^ Importantly, these simulations showed a rather similar trend of ⟨α⟩ vs *Wi* for the different concentrations tested (0 < *c*/c* ≤ 2).

**Figure 4:**
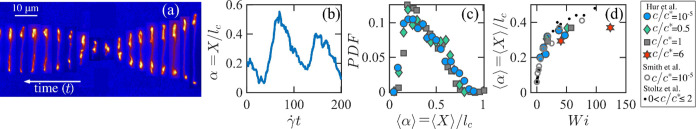
(a) Sequence of snapshots of fluorescent DNA (*l*_*c*_ = 80 μm).^31^ (b) Fractional extension α = *X*/*l*_c_ of DNA as in (a) as a function of the shear strain defined as γ=γ̇t at *Wi* = 12.^31^ (c) Steady-state probability distribution function (PDF) for λ-DNA at various concentrations (see the legend on the far right) as a function of fractional extension at a comparable value of *Wi* in the range 48–60.^32^ (d) Steady-state fractional extension as a function of *Wi* at various concentrations given by the legend on the far right.^32–34^ Image in (a) is adapted from ref 31 with permission from the American Physical Society. Data in (b) are from Schroeder et al.^31^ and data in (c) are from Hur et al.^32^ Data in in (d) are from Hur et al.,^32^ Smith et al.^33^ and Stoltz et al.^34^ Data in (b–d) were extracted from the cited source using WebPlotDigitizer.^18^

Measurements of the infinite-shear viscosity (η_∞_) of λ-DNA at high shear rates, i.e., when the polymer reaches at least transiently a high degree of stretching, have shown a direct proportionality between η_∞_ – η_s_ and polymer concentration *c* (i.e., η_∞_ – η_s_ ∼ *c*), up to concentrations that generate a significant level of entanglement at equilibrium.^36^ This has led to the concept that entangled λ-DNA solutions, and semiflexible polymers in general, can disentangle and stretch at sufficiently high shear rates, thereby reducing interpolymer interactions and acting effectively as if in the dilute regime.^36,37^

For semiflexible polymers that adopt a free-draining configuration, such as the λ-DNA discussed above, substantial stretching can occur in shear flow because each segment of the chain experiences little hydrodynamic shielding. In contrast, flexible polymers, such as commonly used high-*M*_w_ polystyrene or poly­(ethylene oxide), are much less prone to stretch under shear and typically require extensional flows to achieve appreciable deformation measurable by bulk techniques such as birefringence or scattering.^11,38,39^

## Extensional Flow

Extensional flows are ideal for studying the stretching dynamics of macromolecules, as the absence of vorticity allows polymers to reach a steady conformational state with minimal fluctuations in extension, particularly when compared to shear flows, and to accumulate large molecular strains. However, generating extensional flows in polymeric liquids that are truly “rheometric” that is, spatially homogeneous and steady in both the *Eulerian* and *Lagrangian* frames, is notoriously difficult and remains an active area of research.^5,39–46^ Here, we separately discuss investigations of polymer dynamics and interpolymer interactions under the two most commonly used approaches to generate extensional flow in dilute and semidilute polymer solutions: microfluidic and millifluidic devices, and capillary-driven thinning techniques.

## Microfluidic and Millifluidic Extensional Flows

For low-viscosity fluids, specifically designed micro- and millifluidic devices have proven effective in producing flows with a uniform extensional rate across a large region.^5,40–46^ Perhaps the most suitable platforms for extensional rheometry are those based on extensional flows incorporating a stagnation point, such as four-roll mills, opposed jets, and, more recently, cross-slot microfluidic devices and their optimized variants.^5,40–43,46,47^ The advantage of generating stagnation point flows lies in the ability to achieve a zero local flow velocity at the stagnation point while having a finite strain rate. Hence, polymers passing sufficiently close to the stagnation point are exposed to a specific ε̇ for a relatively long residence time *t*_res_ and therefore able to accumulate high macromolecular strain (ε_mol_).

The earliest studies employing stagnation point flows to investigate polymer stretching under extensional flow conditions originated from the Bristol group, with the first qualitative experimental observations dating back to 1971.^50^ In 1978, Pope and Keller used opposed-jet devices to study polystyrene solutions in uniaxial extensional flows (Figure 5(a)).^51^ They identified the onset of the coil-to-stretch transition above a critical extension rate ${\mathrm{\varepsilon ̇}}_{c}$ by observing the emergence of a localized birefringent strand (Figure 5(b)), whose intensity directly correlates with the concentration of aligned polymer segments. Notably, they found that for solutions at *c* ≪ c*, at a fixed extension rate (and presumably a constant *Wi*, given the dilute regime and a concentration independent τ), the birefringence Δ*n* normalized by polymer concentration *c* (i.e., Δ*n*/*c* and referred to as intrinsic birefringence) remained constant. This behavior was attributed to the absence of interpolymer interactions among the highly stretched chains. In contrast, at *c* ≳ c*, they observed a delocalized birefringence pattern that was initially referred as “a strange effect” and in later publication referred to as the “flare” (see Figure 5(c)).^51^ This delocalization of birefringence was associated with the emergence of interpolymer interactions when stretched, a phenomenon that spurred further investigation. Building on the aforementioned work,^51^ more systematic work on polymer solutions at $c\approx O\left(c^{*}\right)$ indicated that the “flare” occurs at a critical extension rate ${\mathrm{\varepsilon ̇}}_{n}>{\mathrm{\varepsilon ̇}}_{c}$ . The occurrence of the flare at $\mathrm{\varepsilon ̇}={\mathrm{\varepsilon ̇}}_{n}$ was interpreted as indicating a time scale $1/{\mathrm{\varepsilon ̇}}_{n}$ below which polymer chains do not have sufficient time to disentangle. Thus, the “flare” was associated with the presence of a transient network that could form at time scales shorter than $1/{\mathrm{\varepsilon ̇}}_{n}$ and at concentrations as low as c*/70.^48,52,53^ The critical concentration for the appearance of the flare was proposed as a more reliable indicator of the onset of interpolymer interactions than the estimate provided by the geometrical argument in eq 1 or similar approaches. It is interesting to compare these observations from the 1980s^48,52,53^ with more recent experiments conducted using an optimized cross-slot device and similar polystyrene solutions at *c* < c* by Haward et al.^49^ The optimized cross-slot geometry produces planar extensional flows that are homogeneous and nearly shear-free over a large region surrounding the stagnation point (Figure 5(d)). In these experiments, it was observed that at sufficiently high *Wi*, the flow transitioned from a steady and symmetric state to an asymmetric and transient one. This change in flow behavior was accompanied by a shift in the birefringence pattern, from a well-defined strand to a characteristic “flare”-like structure (Figure 5(e) and (f), respectively). However, the emergence of a flare-like structure, along with a pronounced change in the flow pattern, was attributed, by Haward et al.,^49^ to the onset of an elastic fluid instability induced by the normal stresses developed during the stretching of the polymer chain rather than the formation of a transient polymer network as previously suggested by the Bristol’s group.^48,52,53^ The interpretation of the observed phenomenon as a fluid instability was supported by applying a universal criterion for the onset of elastic instabilities.^54^ This suggests that the ”flare”-like birefringence patterns reported by the Bristol group may not necessarily reflect interpolymer interactions or the formation of a transient polymer network.

**Figure 5:**
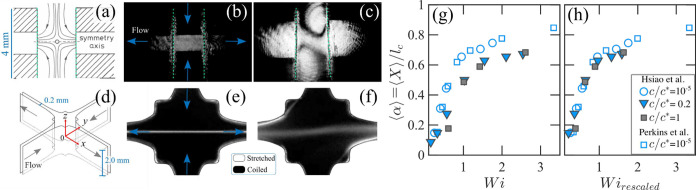
(a) Schematic of the opposed-jet device in suction mode used by the Bristol group.^48^ Birefringence of a polystyrene solution flowing through the opposed-jet device at extension rates below and above those required for the appearance of the flare pattern, shown in (b) and (c), respectively.^48^ (d) Schematic of the optimized cross-slot microfluidic device, referred to as the Optimized-Shape Cross-Slot Extensional Rheometer (OSCER), used by Haward et al.^49^ Birefringence of a polystyrene solution flowing in the OSCER device at relatively low and high *Wi* is shown in (e) and (f), respectively, with flow instability occurring in (f). (g) Averaged fractional extension, ⟨α⟩ = ⟨*X*⟩/*l*_c_, of fluorescently labeled λ-DNA at different concentrations, trapped in a stagnation point extensional flow as a function of *Wi*. Data in (h) display the same data as in (g) with a rescaled *Wi* (*Wi*_rescaled_) for the semidilute solutions. Data in (g) and (h) are from Hsiao et al.^21^ and Perkins et al.^25^ Panels (a–c) are adapted with permission from Elsevier. Panels (e) and (f) are reproduced from Haward et al.^49^ (available under a CC-Creative Commons CC BY license) and converted to black and white from the original version. Data in (g) and (h) were extracted from the cited sources using WebPlotDigitizer.^18^

In the 1980s and 1990s, Leal’s group^55–57^ thoroughly investigated the effect of polymer stretching on flow modification using two- and four-roll mill devices. They reported that above a critical concentration lying below c*, the polymer solution displayed clear flow modification, with the extension rate falling below the Newtonian expectation. This critical concentration, $c_{h}^{*}$ , was proposed to mark the upper limit of a new regime, termed the *ultradilute regime* $\left(c_{h}^{*}<c^{*}\right)$ , and was associated with the concentration at which fully stretched polymers begin to hydrodynamically interact. The absence of flow modification in the ultradilute regime (i.e., $c<c_{h}^{*}$ ) was attributed to the fact that interpolymer interactions remain negligible regardless of the degree of polymer stretching.^57^ According to Ng et al.,^56^ $c_{h}^{*}$ can be estimated analogously to c* using eq 1 by considering the volume occupied by a polymer as that of a sphere with radius *l*_c_/2, so that $\phi_{\mathrm{sph}}\equiv \pi l_{c}^{3}/6$ . The general concept proposed is that, for $c<c_{h}^{*}$ , each stretched polymer remains sufficiently distant from the others, so that local flow perturbations do not influence neighboring chains and the polymers behave as hydrodynamically independent. In contrast, at $c≳c_{h}^{*}$ , the flow perturbations caused by a stretched polymer influence the neighboring polymers. The idea that highly stretched polymers begin to hydrodynamically interact at a concentration $c_{h}^{*}$ , corresponding to the point where the average interpolymer spacing becomes comparable to the polymer half-contour length (*l*_c_/2), is consistent with Batchelor’s theory for suspensions of elongated particles.^58,59^ Another notable insight proposed by the Leal group based on birefringence measurements was that while polymers in the ultradilute regime reach their maximum fractional extension as *Wi* → ∞, those in the range $c_{h}^{*}<c<c^{*}$ may not.^57^ This behavior was interpreted in terms of polymer–polymer interactions that limit full extension, although the authors explicitly noted that it was unclear why such interactions should produce this effect. It is important to note that, in general for polymers with some degree of flexibility $c_{h}^{*}<c^{*}$ , for instance, in the case of λ-DNA, $c^{*}/c_{h}^{*}\approx O\left(10^{3}\right)$ . Unlike flexible polymers, such as those studied by Leal’s group,^55–57^ which exhibit flow modification at concentrations well below c*, semiflexible polymer solutions have shown no significant deviation from Newtonian flow behavior even at concentrations above c*.^11,60,61^ This suggests that hydrodynamic interactions between stretched polymers alone are insufficient to induce flow modification, and that polymer extensibility appears to play a key role in promoting such changes. However, the precise manner by which polymer extensibility influences flow-induced modifications remains unclear, and it is still unknown how, to what extent, and through which physical mechanisms the nature of intra- and interpolymer interactions affects the resulting flow response. For instance, it would be of particular interest to understand how different dominants modes of intra- and interpolymer interactions, such as those distinguishing polyelectrolytes from neutral polymer chains, impact these flow-induced modifications.

A major advance in understanding polymer behavior under extensional flow came in 1997 with the seminal work of Perkins and co-workers, who directly observed single DNA molecules in the ultradilute regime trapped in a stagnation point extensional flow.^25^ Relevant to this viewpoint, their study demonstrated: (*i*) polymer chains undergo a coil-to-stretch transition at *Wi* ≈ 0.5, but require sufficient accumulated strain to reach full extension; (*ii*) distinct conformational states (e.g., dumbbell, kinked, halfdumbbell, and folded) with differing dynamics, despite identical ε̇ and *t*_res_, can coexist, a phenomenon referred to as molecular individualism by De Gennes.^62^ A direct follow-up to Perkins’ work was carried out by Hsiao et al.,^21^ who investigated DNA dynamics trapped in a stagnation point extensional flow under ultradilute conditions (as for Perkins’ work^25^) and compared them with those observed in the semidilute regime. From steady-state fractional extension measurements of single λ-DNA molecules, Hsiao et al. observed a lower degree of extension for nominally dilute and semidilute solutions at *c* = 0.2c* and *c* = c* compared to the ultradilute case at *c* = 10^–5^ c* (Figure 5(g)). A key finding of their work was that the steady-state fractional extension across the ultradilute, dilute, and semidilute regimes exhibited self-similar behavior, as demonstrated by the collapse of the data onto a single master curve when the *Wi* for the semidilute solutions was rescaled (*Wi*_rescaled_) (Figure 5(h)). This *Wi*_rescaled_ was obtained by identifying the critical *Wi* corresponding to the coil–to–stretch transition in the *c* = 0.2c* and *c* = c* DNA solutions. This self-similar behavior may indicate that the average stretching behavior is analogous across different concentrations, provided the critical *Wi* for the onset of stretching is properly accounted for. Perhaps surprisingly, similar experiments in shear flows showed a good collapse of the fractional extension without the need to rescale *Wi* (shown previously in Figure 4(d)).^32^ Additionally, Hsiao et al.^32^ noted that the transient stretching dynamics of DNA in dilute and semidilute solutions become similar at high *Wi*. Taken together, these single-molecule results indicate that individual polymer chains, when highly stretched may experience a “dilute-like” environment, at least within the concentration range investigated.

## Capillary-Driven Extensional Flows

Techniques based on the capillary-driven thinning of fluid filaments have been widely employed to study polymer dynamics under extensional deformation.^4,41,63^ These methods rely on the formation of a sufficiently thin liquid bridge, in which surface tension drives the spontaneous thinning of the filament, without the need for external forcing, thereby generating a uniaxial extensional flow. A variety of experimental protocols have been developed to initiate this self-thinning process (e.g.,^64–66^ ), all of which exploit the same fundamental physical principle. For highly viscous Newtonian fluids, the thinning dynamics are governed by a balance between viscous and capillary stresses, commonly referred to as the visco-capillary (VC) balance.^63^ In this regime, the filament diameter at the neck of the filament *D* decreases monotonically over time, and the corresponding extension rate ε̇ increases as thinning progresses (Figure 6(a–c)). When a small amount of polymer (as little as a few ppm) is dissolved in a viscous solvent, elastic stresses from the stretched polymer chains begin to play a role. In such polymer solutions, after an initial VC regime (when inertia is negligible), the filament evolution enters an elastocapillary (EC) regime.^63,64^ In this regime, a high–aspect-ratio cylindrical filament forms, markedly different from its Newtonian counterpart (see Figure 6(a) vs (d)), and the filament diameter decreases exponentially with time *t*, according to the time constant τ_*EC*_ as *D* ∼ exp­(−*t*/τ_EC_) (Figure 6(e)). An important consequence of this exponential thinning is the generation of a steady-state extension rate at the neck of the filament, which may or may not be sustained long enough for the polymer chains to reach an effectively time-independent conformation (Figure 6(f)). Considerable theoretical, simulation, and experimental effort is currently focused on uncovering the physical origin of the EC regime.^67–72^ The classical interpretation based on the Oldroyd-B constitutive model is that the longest polymer relaxation time τ is intrinsically related to the time constant τ_EC_ and τ ≡ τ_EC_/3.^63^

**Figure 6:**
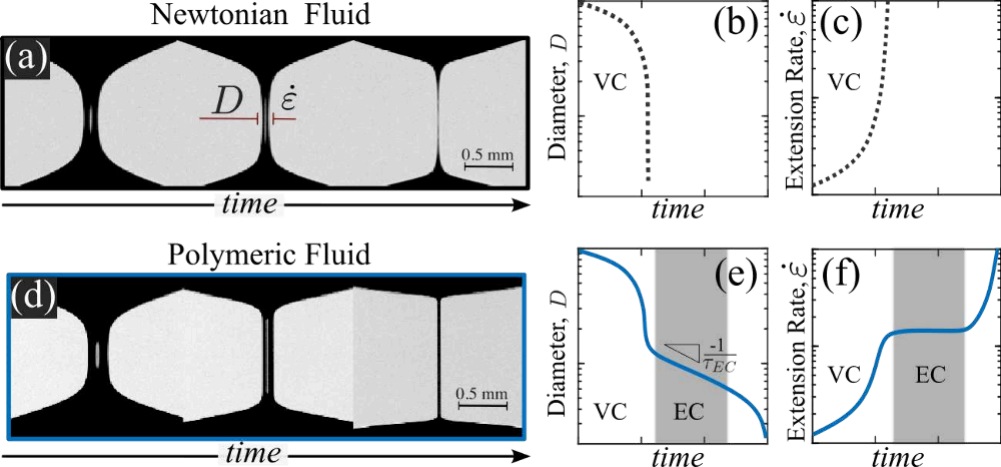
(a) Snapshots of the self-thinning of a viscous Newtonian fluid (glycerol), with schematics showing the evolution of the filament diameter *D* and the extensional rate at the filament neck ε̇ over time in (b) and (c), respectively. (d) Snapshots of the self-thinning of a λ-DNA solution (as in ref 70), with corresponding schematics of the evolution of *D* and ε̇ over time shown in (e) and (f), respectively. Note that the graphs in (b), (c), (e), and (f) are plotted in semilogarithmic representation (logarithmic *y*-axis).

Comparison of τ_EC_/3 retrieved via capillary-driven extensional flows with the relaxation time τ obtained from equilibrium rheometric measurements has, in some cases, confirmed the expected relationship τ ≈ τ_EC_/3.^63,70,71,73^ However, in many other cases, this equivalence has been shown to not hold, i.e., τ ≠ τ_EC_/3.^64,69,70,73,74^ This discrepancy has been generally attributed to conformational differences between polymers at equilibrium and those in a highly stretched state under strong extensional flow. Based on this argument, it has become common to refer to τ_EC_/3 as a distinct material property, known as the “extensional relaxation time”, to distinguish it from τ obtained from shear measurements at equilibrium (also sometimes referred to as the “shear relaxation time”). In line with the central focus of this perspective, several studies discuss the distinction between the time scale obtained from capillary-driven thinning techniques (τ_EC_) and the relaxation time τ measured by conventional rheometry, attributing their differences to interpolymer interactions that emerge as polymers acquire a stretched configuration. Clasen et al.,^64^ conducted one of the most influential studies addressing the origin of the discrepancy between τ and τ_EC_. In their paper, they reported an increasing τ_EC_ with concentration even for *c* < c*, a surprising result, since if τ_EC_/3 effectively corresponds to the longest relaxation time τ, one would expect it to remain independent of concentration in this regime. In fact, both theoretical predictions and rheometric measurements on the same polymer solutions used for capillary thinning experiments indicated that τ remains approximately constant up to c*. These results were interpreted in terms of emerging intermolecular interactions between stretched polymers at concentrations well below c*, leading to an increase in τ_EC_ and thus in the inferred relaxation time in extension, as if the solutions were more concentrated. This phenomenon was referred to as *self-concentration*.^64^

Prabhakar et al.^75^ interpreted Clasen’s experimental results^64^ by proposing a model that accounts for conformation- and concentration-dependent hydrodynamic interactions, ultimately leading to τ_EC_/3 varying with concentration in a manner distinct from τ. According to this model, referred to as the C2D2 model,^30,76^ at concentrations greater than $c_{h}^{*}$ , there is another critical concentration, here referred to as the stretched overlap concentration $c_{s}^{*}$ , corresponding to the concentration at which fully stretched polymer chains begin to overlap. In the following sections, we introduce several approaches to estimate $c_{s}^{*}$ , and we distinguish between them by adding a second subscript to $c_{s}^{*}$ (e.g., $c_{s,P}^{*}$ ). The $c_{s}^{*}$ defined by Prabhakar et al.,^75^ here denoted as $c_{s,P}^{*}$ , effectively accounts for the volume of a cylinder, ϕ_cyl_, with length *l*_c_ and a cross-sectional radius *r* taken as the equilibrium size of the polymer *X*_0_ (i.e., *r* = *X*_0_), such that $c_{s,P}^{*}∼1/\phi_{\mathrm{cyl}}∼1/\left(l_{c}X_{0}^{2}\right)$ . Here, *r* represents the transverse fluctuation length scale of the stretched polymer. According to the C2D2 model, for $c≲c_{h}^{*}$ , interpolymer interactions are negligible, regardless of the degree of flow-induced stretching. For $c_{h}^{*}≲c≲c_{s,P}^{*}$ , chain stretching enhances interpolymer hydrodynamic interactions relative to equilibrium, giving rise to a self-concentration effect. In the regime $c_{s,P}^{*}≲c≲c^{*}$ , the influence of flow-induced hydrodynamic interactions among stretched chains weakens relative to equilibrium, leading to a self-dilution effect. Finally, for *c* ≫ c*, stretching no longer alters hydrodynamic screening compared with equilibrium conditions. The C2D2 model successfully captured the concentration dependence of τ_EC_ and, more recently, has also been able to explain the dependence of τ_EC_ on the initial aspect ratio of the fluid filament prior to initiating the self-thinning protocol.^72,76^ Overall, the C2D2 model predicts that polymer chain stretching under flow causes interpolymer interactions to depend on concentration in a nontrivial manner.

To explain the concentration dependence of τ_EC_ in capillary-driven experiments even in nominally dilute polymer solutions, Dinic et al, invoked the concept of the stretched overlap concentration $c_{s}^{*}$ .^4,77^ As before, the physical meaning of $c_{s}^{*}$ is to describe the polymer concentration at which fully stretched polymers interact via direct overlap. The $c_{s}^{*}$ defined by Dinic et al., here denoted as $c_{s,D}^{*}$ , is based on the Doi–Edwards framework, which defines equilibrium concentration regimes for rigid rod-like polymers or colloids.^2^ Doi and Edwards identified the critical overlap concentration as $c^{*}∼1/l_{c}^{3}$ and the threshold for the concentrated regime as $c^{**}∼1/\left(bl_{c}^{2}\right)$ , where *b* is the rod diameter. According to this approach, rod-like polymers are isolated and noninteracting for *c* < c*, whereas interpolymer interactions become significant at *c* ≳ c*. This onset of interpolymer interactions at *c* ≳ c* is reflected in the emergence of a different scaling law for the polymeric viscosity, η_p_(*c*), and in the relaxation time of the rods, which became concentration-dependent, as widely demonstrated experimentally (e.g.,^78–80^ ). Dinic et al. formulates the stretched overlap concentration as $c_{s,D}^{*}∼1/\left(bl_{c}^{2}\right)$ , analogously to the onset of the concentrated regime c** defined by Doi and Edwards.^2^ However, the rationale for selecting c** as a reference for the estimation of $c_{s}^{*}$ , is not discussed.^4,77^ The key interpretation offered by Dinic et al. is that highly stretched polymers overlap more readily than at equilibrium, implying a reduced effective overlap concentration for stretched chains, $c_{s,D}^{*}$ , compared to c*, and thus a concentration-dependent extensional relaxation time, τ_EC_/3, even in nominally dilute solutions.

Recent experiments by Calabrese et al., highlighted the critical role of the *M*_w_-distribution in determining τ_EC_.^69^ By blending dilute polystyrene solutions composed of significantly different *M*_w_ fractions, the authors demonstrated that, depending on concentration, different portions of the *M*_w_-distribution dictate the EC regime. This behavior was conceptualized as follows: as filament thinning progresses in the VC regime and the strain rate ε̇ increases, progressively shorter chains within the distribution begin to stretch, since smaller chains require higher ε̇ to undergo coil-to-stretch transitions. Eventually, the cumulative elastic stress generated by the stretched chains becomes sufficient to balance the viscous stress of the solvent, initiating the EC balance. Thus, at very low concentrations (see Figure 7(a,b)), as self-thinning begins, even stretching of the entire molecular weight distribution is insufficient to generate the elastic stress required to overcome the viscous stresses. As a result, the fluid continues to thin similarly to a Newtonian fluid (Figure 7(a, b)). At moderate concentrations, most of the distribution must stretch in order to establish the EC balance in the low-*M*_w_ tail (Figure 7(c,d)). At high concentrations, only the longest chains (in the high-*M*_w_ tail) contribute to the elastic stress, while a large portion of the lower-*M*_w_ chains remains unaffected by the flow (Figure 7(e,f)). This occurs because the strain rate in the EC regime is not sufficient to induce stretching of these shorter chains. Thus, as the polymer concentration increases, the EC regime becomes increasingly dominated by longer chains, ultimately leading to a value of τ_EC_ that increases with concentration, even in nominally dilute solutions. By simply accounting for chain polydispersity, an inherent feature of polymer solutions, this concept of progressive chain stretching suggests that, even in the absence of interpolymer interactions, τ_EC_ is expected to increase with concentration in dilute solutions. Within this framework, the reported τ_EC_ increasing with concentration even in nominally dilute solutions by Clasen et al.,^64^ and Dinic et al.,^77^ may simply reflect that the EC regime becomes governed by progressively larger polymers in the distribution as concentration increases, rather than arising directly from emergent interpolymer interactions occurring under flow.

**Figure 7:**
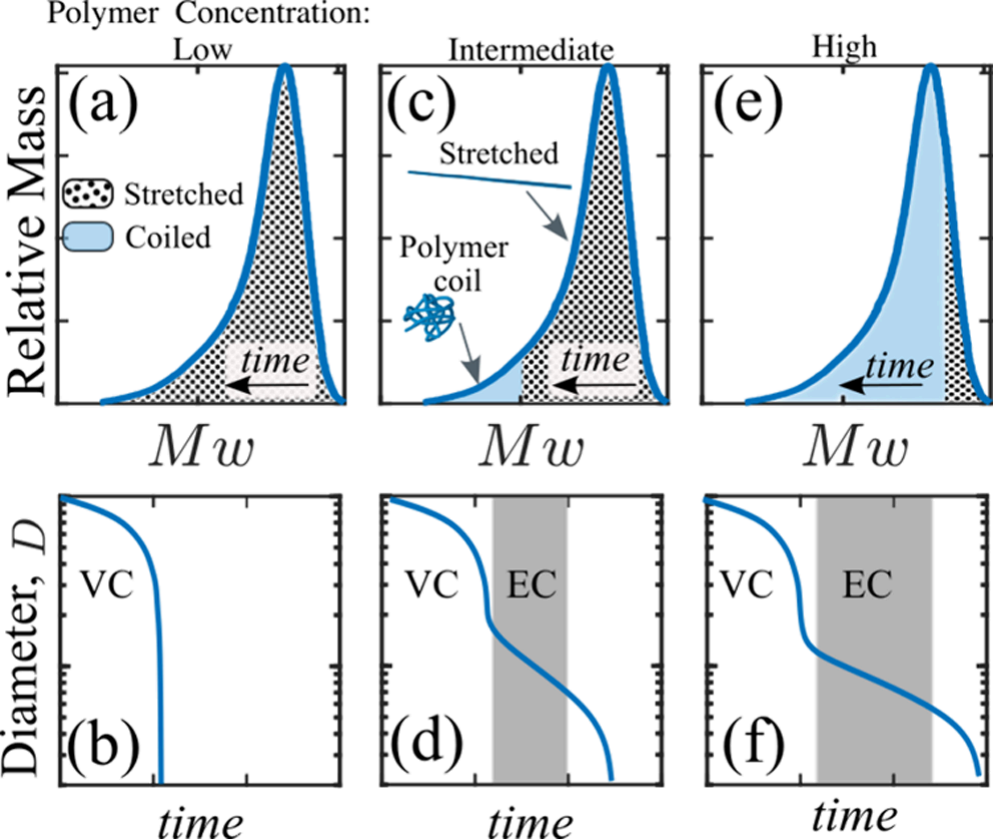
Schematics of progressive chain stretching at three distinct polymer concentrations (low, intermediate, and high). (a, c, e) illustrate the relative mass fractions of stretched (dotted pattern) and coiled polymers within the molecular weight distribution, while (b, d, f) show the corresponding filament-thinning dynamics. (a, b) The low-concentration regime, where polymers across the molecular weight distribution are fully stretched but generate insufficient elastic stress to overcome the viscous stress of the solvent; as a result, filament thinning follows the VC balance. (c, d) An intermediate concentration regime, where high-*M*_w_ polymers stretch and generate sufficient elastic stress to establish an EC balance and a measurable τ_EC_. However, the extension rate set during the EC balance is too low to stretch the low-*M*_w_ polymers, which remain coiled. (e, f) represent a relatively high-concentration regime, in which only polymers in the high-*M*_w_ tail of the distribution stretch and dominate the EC balance, while most chains in the distribution remain coiled.

It is worth noting that all previously discussed frameworks addressing the concentration dependence of τ_EC_ in the dilute regime rely on the assumption that τ_EC_ is directly related to the longest polymer relaxation time, typically expressed as τ ≈ τ_EC_/3. As such, these frameworks ultimately interpret variations in τ_EC_ as changes in an intrinsic material property τ rather than treating τ_EC_ as an independent time scale emerging from capillary-driven thinning. However, it is important to recognize that if this assumption is violated, the validity of these frameworks, and the interpretations they support, such as the role of interpolymer interactions under stretching, may be fundamentally compromised.

In a recent study, Nardone et al. investigated DNA solutions undergoing capillary-driven thinning.^74^ While still extracting τ_EC_ from their filament thinning profile, they interpreted τ_EC_ as an independent time scale emerging from capillary-driven thinning.^74^ They hypothesized that, under strong extensional flows and for moderate concentrations in the range *c* ≲ 10c*, interpolymer interactions among stretched chains may be less significant than at equilibrium. This hypothesis was based on two key experiments: (*i*) The measured τ_EC_ for two distinct monodisperse DNA samples followed a monotonic power-law increase with concentration, without any noticeable deviation in the power-law exponent at *c* ≈ c* or *c* ≈ 5c*, the latter roughly corresponding to the onset of the entanglement regime (see Figure 8(a)). We note that at these two critical concentrations, as previously shown in Figure 2, the polymeric viscosity η_p_ and the relaxation time τ display a different scaling with concentration due to enhanced chain confinement. In contrast, the τ_EC_ trend remained largely insensitive to the changes that occur under equilibrium conditions. (*ii*) In a second experiment, the authors blended two monodisperse DNA species: λ-DNA at a fixed concentration (*c* ≈ 5c*) with a variable concentration of higher *M*_w_ T4-DNA. Remarkably, the time scale τ_EC_ of the blend was fairly well described by the additive contribution of the τ_EC_ values of the two individual DNA solutions (Figure 8(b)). This additive behavior suggested minimal interpolymer interactions, despite the fact that the background λ-DNA was already at a concentration close to the entangled regime.^74^ The authors suggested, following Dakhil et al.,^36,37^ that the highly stretched conformation of DNA leads to an occupied volume that is that of a cylinder of length *l*_c_ and cross-sectional radius *r*, given by ϕ_cyl_ = 2π*r* ^2^ *l*_c_. The cross-sectional radius of the cylinder, *r*, representing the transverse fluctuations of the polymer chain, was assumed to be smaller than the polymer’s persistence length when the chain is highly stretched at *Wi* ≫ 0.5. Using ϕ_cyl_ in eq 1 instead of ϕ_sph_, Nardone et al. defined an overlap concentration for fully stretched chains as $c_{s,N}^{*}=\left(M_{w}\phi_{\mathrm{pf}}\right)/\left(\phi_{\mathrm{cyl}}N_{A}\right)$ .^74^ Interestingly, since both *M*_w_ ∼ *l*_c_ and ϕ_cyl_ ∼ *l*_c_, $c_{s,N}^{*}$ turns out to be independent of *M*_w_ for a given polymer. This prediction is particularly interesting to test in future work. Importantly, the approach used by Nardone et al.^74^ leads to $c_{s,N}^{*}/c^{*}>1$ , in contrast to that of both Prabhakar et al., and Dinic et al., where $c_{s,P}^{*}/c^{*}<1$ and $c_{s,D}^{*}/c^{*}<1$ , respectively.^75,77^

**Figure 8:**
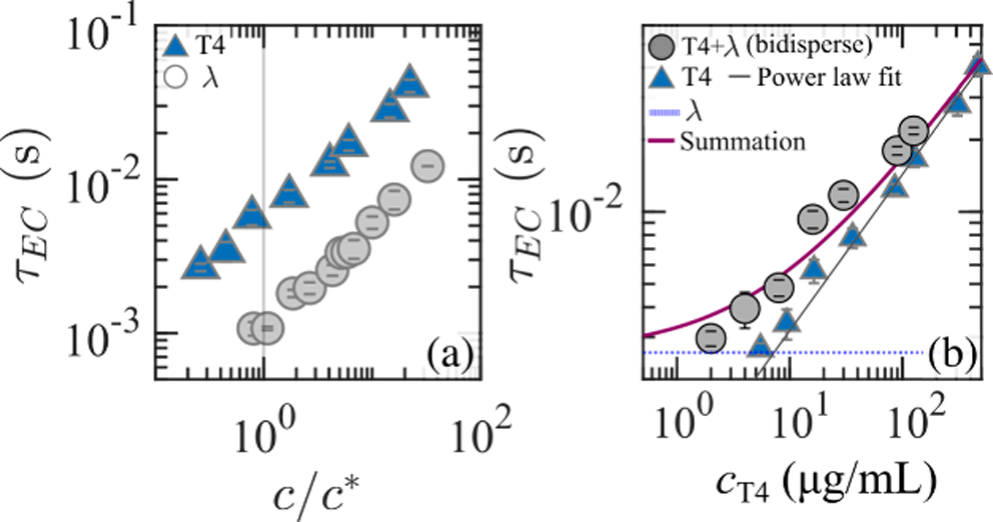
(a) Elastocapillary time scale, τ_EC_, as a function of normalized concentration for λ- and T4-DNA, showing an analogous power-law dependence of τ_EC_ on concentration. (b) τ_EC_ as a function of T4-DNA concentration (*c*_T4_) for monodisperse T4 (blue triangles) and bidisperse solutions (gray circles) consisting of a constant λ-DNA concentration while varying the T4 concentration. The τ_EC_ of the constant background λ-DNA is indicated by the blue dotted line, and the summation model, which combines the contributions of τ_EC_ from pure T4 and pure λ-DNA, is shown as the dark red solid line.

## Summary, Perspective, and Outlook

By comparing different studies side by side, we encountered various proposed critical concentrations at which polymers begin to interact when fully stretched either hydrodynamically (i.e., $c_{h}^{*}$ ) or by overlapping (i.e., $c_{s}^{*}\equiv c_{s,P}^{*}$ , $c_{s}^{*}\equiv c_{s,D}^{*}$ , $c_{s}^{*}\equiv c_{s,N}^{*}$ , depending on the estimation procedure). Here, we discuss their dependence on the extent of polymer stretching, rather than restricting the discussion to the limiting case of fully stretched chains. This approach is particularly useful for describing the behavior of polymer solutions in extensional flows as *Wi* increases and the stretching of the chain becomes more pronounced. Following the approach of Prabhakar et al.,^75^ we construct a polymer stretching–concentration diagram to illustrate how these critical concentrations scale with the extent of polymer stretching (Figure 9). The degree of polymer stretching is parametrized by the fractional extension α = *X*/*l*_c_, with the equilibrium fractional extension given by α_0_ = *X*_0_/*l*_c_. We assume that $c^{*}∼R_{g}^{-3}∼{\left(X_{0}\right)}^{-3}$ . In Figure 9, α_0_ is set to 0.1, a representative rounded value consistent with that of λ-DNA.

**Figure 9:**
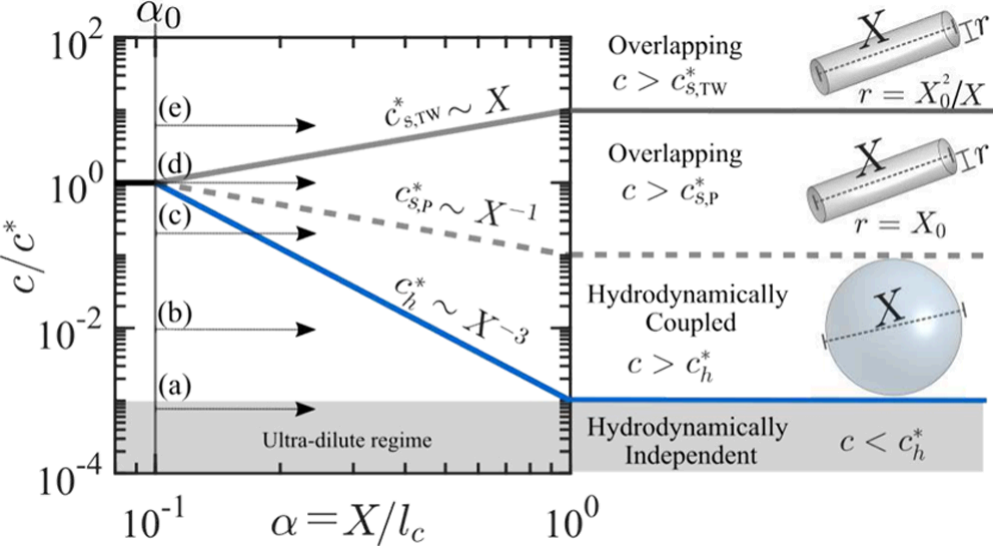
Concentration–stretching diagram. The polymer concentration is expressed as *c*/c*, and the stretching is parametrized as the fractional extension α = *X*/*l*_c_, with the equilibrium fractional extension taken as α_0_ = 0.1. The lines indicate the thresholds above which interpolymer interactions are expected according to different theoretical frameworks.

As established by Leal and co-workers,^55–59^ based on Batchelor’s theory for rigid rods, for $c>c_{h}^{*}$ , hydrodynamic interactions are expected due to overlapping flow disturbances. For fully stretched chains, $c_{h}^{*}$ is based on the hydrodynamically perturbed volume of a sphere of radius *l*_c_/2, yielding $c_{h}^{*}∼1/\phi_{\mathrm{sph}}∼1/l_{c}^{3}$ . As *Wi* increases and the end-to-end distance increases, $c_{h}^{*}∼1/X^{3}$ , leading to the ratio $c_{h}^{*}/c^{*}∼X^{-3}∼\alpha^{-3}$ (see blue continuous line in Figure 9). Thus, as α increases beyond the equilibrium value α_0_, $c_{h}^{*}$ decreases cubically with fractional extension. The same scaling for the onset of hydrodynamically interacting polymers was also given by Prabhakar et al.^75^

At concentrations greater than $c_{h}^{*}$ , $c_{s}^{*}$ marks the onset of overlap between stretched chains through direct contacts. Prabhakar et al. effectively model the stretched polymer as an object with cylindrical geometry, whose effective volume is determined by its extension *X* and a constant cross-sectional radius *r*, taken to be the equilibrium polymer size *X*_0_ (i.e., *r* = *X*_0_). This leads to the scaling $c_{s,P}^{*}/c^{*}∼X^{-1}$ (see gray dashed line in Figure 9).^30^ However, when *Wi* exceeds 0.5, the polymer becomes increasingly aligned and explores progressively smaller distances perpendicular to its principal axis. Consequently, for *Wi* > 0.5, the transverse fluctuation length scale *r* is expected to decrease from its initial value, *r* = *X*_0_. Here, we illustrate the effect of this reduction in transverse fluctuations by assuming an inverse proportionality between *r* and the end-to-end distance *X* such that $r=X_{0}^{2}/X$ . This formulation ensures that *r* = *X*_0_ at equilibrium and that *r* decreases continuously with increasing chain stretch, reaching a minimum at full extension. This approach provides only an indicative estimate but serves to capture the essential concept. Substituting $r=X_{0}^{2}/X$ into ϕ_cyl_ leads to a definition of the stretched overlap concentration proposed in this work (hereafter denoted by the subscript TW) as $c_{s,TW}^{*}∼1/\left(Xr^{2}\right)$ . This yields $c_{s,\mathrm{TW}}^{*}∼X/X_{0}^{4}$ and, consequently, $c_{s,\mathrm{TW}}^{*}/c^{*}∼X$ , shown as the gray solid line in Figure 9. In the limiting case of a fully stretched polymer, $c_{s,\mathrm{TW}}^{*}$ is analogous to that proposed by Nardone et al.,^74^ as both approaches describe the polymer volume as that of a cylinder with a transverse fluctuation length scale *r* < *X*_0_.

Neglecting differences in the packing fraction ϕ_pf_ between stretched and coiled chains, the distinct critical concentrations can be approximated as follows. Onset of hydrodynamic interactions based on Ng et al.:^56^

2 $$\frac{c_{h}^{*}}{c^{*}}=\frac{\alpha_{0}^{3}l_{c}^{3}}{X^{3}}\underset{\mathrm{for}X=l_{c}}{\to }\alpha_{0}^{3}$$

Onset of overlap for flow-stretched polymers based on Prabhakar et al.^75^ (i.e., $c_{s}^{*}\equiv c_{s,P}^{*}$ ):

3 $$\frac{c_{s,P}^{*}}{c^{*}}=\frac{\alpha_{0}l_{c}}{X}\underset{\mathrm{for}X=l_{c}}{\to }\alpha_{0}$$

Onset of chain overlap for flow-stretched polymers in this work (based on Nardone et al., and Dakhil et al.,^36,37,74^ i.e., $c_{s}^{*}\equiv c_{s,\mathrm{TW}}^{*}$ ):

4 $$\frac{c_{s,\mathrm{TW}}^{*}}{c^{*}}=\frac{X}{\alpha_{0}l_{c}}\underset{\mathrm{for}X=l_{c}}{\to }\frac{1}{\alpha_{0}}$$

Based on the polymer stretching–concentration diagram constructed from the equations above, and using α_0_ = 0.1 as a representative value for λ-DNA, we can identify distinct regimes of polymer interactions, as suggested by the different frameworks (i.e., $c_{h}^{*}$ , $c_{s,P}^{*}$ , $c_{s,\mathrm{TW}}^{*}$ ). The power-law boundaries plotted in the diagram delineate the limits of each interaction regime (Figure 9). For a given polymer stretching α, concentrations above these boundaries indicate the onset of specific interpolymer interaction modes, depending on the adopted framework.

At very low polymer concentrations $\left(c/c^{*}<O\left(10^{-3}\right)\right)$ , increasing polymer stretching (i.e., following arrow (a)) does not lead to entry into any additional interaction regime. This implies that for $c/c^{*}<O\left(10^{-3}\right)$ , polymers can be considered *ultradilute*, as they remain noninteracting regardless of the extent of stretching, α. At slightly higher concentrations, though still in the nominally dilute regime, for example, *c*/c* = 10^–2^ (following arrow (b)), results in a crossover with $c_{h}^{*}$ , but only above a critical threshold of fractional extension. This suggests that polymers can begin to interact hydrodynamically under flow once they are sufficiently stretched. At *c*/c* = 0.2, increasing flow-induced polymer stretching (arrow (c)) leads to two successive crossovers: first with $c_{h}^{*}$ and then with $c_{s,P}^{*}$ . This indicates that, above a certain stretch level, polymer chains may interact both hydrodynamically and through direct overlap. At *c*/c* = 1, increasing flow-induced polymer stretching (arrow (d)) does not lead to a crossover with any critical concentration. However, the system is already in a regime where polymers may interact hydrodynamically and via direct overlap according to $c_{s,P}^{*}$ (note that arrow (d) lies above $c_{h}^{*}$ and $c_{s,P}^{*}$ ). At *c*/c* = 5 (arrow (e)), polymers overlap already at equilibrium, and as the stretch progresses, the system remains in the overlapping regime until it crosses a threshold fractional extension defined by $c_{s,\mathrm{TW}}^{*}$ . Beyond this point of fractional extension, polymers exit the overlap regime defined by $c_{s,\mathrm{TW}}^{*}$ , and the effect of interchain overlap is predicted to diminish. This is because, according to $c_{s,\mathrm{TW}}^{*}$ , a sufficiently stretched polymer occupies a cylindrical volume that is smaller compared to the spherical volume it occupies at equilibrium. However, according to $c_{s,P}^{*}$ the polymers are already highly overlapped. This emphasizes that the predicted physical scenario depends strongly on how the volume of a stretched polymer is estimated when determining $c_{s}^{*}$ . For instance, assuming a constant transverse fluctuation length scale *r*, as in $c_{s,P}^{*}$ , predicts increasing overlap with stretching, whereas accounting for a decreasing *r* with stretching, as in $c_{s,\mathrm{TW}}^{*}$ , leads to the opposite trend, with the overlap of stretched chains decreasing as the polymer stretches. Furthermore, we note that the expressions provided in eqs 2, 3, and 4 do not account for the role of electron clouds and their flow-induced deformation as a function of polymer stretching, which is particularly relevant for salt-free polyelectrolytes. For this reason, these equations should be regarded primarily as conceptual descriptors and starting points for future experimental investigations, rather than as detailed, predictive solutions.

Now that these regimes have been distinguished, it is worth considering when such interactions might be experimentally detectable. The transition into a hydrodynamically interacting regime with chain extension, such as that illustrated by arrow (b) in Figure 9, may not be accompanied by a pronounced change in microstructure, since polymer chains do not physically interact but are thought to interact through long-range hydrodynamic perturbations. On the other hand, hydrodynamic interactions result in an overall increase in the frictional coefficient per chain.^75^ Therefore, the onset of these interactions, such as the crossover indicated by arrow (b) in Figure 9, may in principle be detectable in rheological measurements. In particular, they could be probed in experiments where the extensional stress is measured as a function of the applied ε̇ , thereby modulating the degree of chain extension. While such experiments could offer valuable insights into the onset of intermolecular interactions under flow, they remain technically challenging, particularly due to the difficulty of applying steady-state extensional deformation while accurately measuring the typically small extensional stresses in highly dilute polymer solutions. In contrast, when stretched polymers physically overlap $\left(c>c_{s}^{*}\right)$ , probing the microstructural evolution via birefringence, scattering, or single-molecule experiments may provide an effective means to infer intermolecular interactions mediated by contacts between stretched polymers.

While the stretching–concentration diagram offers only a qualitative framework, it suggests a possible interpretation of the experimental results of Hsiao et al., shown previously in Figure 5(g,h).^21^ Their data demonstrated that, although the *Wi* for the onset of the coil-to-stretch transition depended on polymer concentration, the overall chain dynamics collapsed onto a universal curve when a rescaled *Wi* was used (Figure 5(h)). By mapping the DNA concentrations used in Hsiao’s experiments onto the stretching–concentration diagram, at *c*/c* = 0.2 (arrow (c)) and *c*/c* = 1.0 (arrow (d)), the systems lie below the proposed overlap threshold $c_{s,\mathrm{TW}}^{*}$ , irrespective of the degree of polymer stretching. This may explain why, even at *c*/c* = 1, the polymer chains behaved in a “dilute-like” manner at high *Wi*, yielding fractional extension data that could be collapsed onto a single master curve. It would be interesting in future work to map the stretching of λ-DNA, as in Hsiao et al.,^21^ over a large concentration range (up to concentrations significantly exceeding $c_{s,\mathrm{TW}}^{*}$ ) to determine when the self-similar behavior of the fractional extension as a function of *Wi* breaks down.

We have revisited seminal experimental studies on the emergence of interpolymer interactions under flow and compared them in light of more recent interpretations. Interestingly, different experiments and theoretical approaches have suggested distinct modalities of interaction for flow-stretched polymer chains, as well as different thresholds for their onset. This highlights current challenges and open questions that call for further theoretical, experimental, and combined investigations. From our analysis, clear avenues for future work emerge. In particular, an immediate step is to test the proposed scalings for $c_{s}^{*}$ against experiments, as well as to clarify how interpolymer interactions between flow-stretched chains differ between neutral and charged polymers. Overall, understanding interpolymer interactions under flow will be crucial for developing robust constitutive models that incorporate flow-dependent interpolymer interactions and more accurately capture a wide range of flow scenarios.
